# Clinical Implications of the NICE 2015 Criteria for Gestational Diabetes Mellitus

**DOI:** 10.3390/jcm7100376

**Published:** 2018-10-22

**Authors:** Meena Bhatia, Lucy H. Mackillop, Katy Bartlett, Lise Loerup, Yvonne Kenworthy, Jonathan C. Levy, Andrew J. Farmer, Carmelo Velardo, Lionel Tarassenko, Jane E. Hirst

**Affiliations:** 1 Oxford University Hospitals NHS Foundation Trust, Headington OX3 9DU, UK; Lucy.mackillop@ouh.nhs.uk (L.H.M.); Katy.bartlett@ouh.nhs.uk (K.B.); 2 Nuffield Department of Women’s Reproductive Health, University of Oxford, Oxford OX3 9DU, UK; yvonne.kenworthy@cardiov.ox.ac.uk (Y.K.); Jane.hirst@wrh.ox.ac.uk (J.E.H.); 3 Institute of Biomedical Engineering, University of Oxford, Oxford OX3 7DQ, UK; Lise@loerup.no (L.L.); carmelo.velardo@eng.ox.ac.uk (C.V.); lionel.tarassenko@eng.ox.ac.uk (L.T.); 4 The Oxford Centre for Diabetes, Endocrinology and Metabolism, Oxford University Hospitals NHS Trust, Oxford OX3 7LE, UK; Jonathan.levy@ocdem.ox.ac.uk; 5 Nuffield Department of Primary Care Health Sciences, University of Oxford, Oxford OX2 6GG, UK; Andrew.farmer@phc.ox.ac.uk

**Keywords:** gestational diabetes mellitus, NICE 2015 guidelines, IADPSG guidelines

## Abstract

Background: In response to concerns that the International Association of Diabetes in Pregnancy Study Group (IADPSG) criteria labeled too many women with gestational diabetes mellitus (GDM) without evidence of clinical or economic benefit, NICE recommended a change in diagnostic criteria in 2015. Aim: To compare diabetes associated maternal and neonatal complications in pregnancies complicated by GDM diagnosed using IADPSG criteria only, to those with GDM diagnosed using both IADPSG and NICE 2015 criteria. GDM screening was risk factor based. Methods: This was a secondary analysis of a trial of women with GDM diagnosed by the IADPSG criteria (fasting blood glucose (BG) ≥ 5.1 mmol/L, 1 h ≥ 10.0 mmol/L and 2 h ≥ 8.5 mmol/L). Outcomes were compared for two groups: NICE + IADPSG defined as those with GDM diagnosed by both the NICE 2015 and IADPSG criteria (fasting BG ≥ 5.6 mmol/L, 2 h ≥ 8.5 mmol/L); and IADPSG-ONLY (fasting BG 5.1 mmol/L to 5.5 mmol/L, and/or 1-hour ≥10.0 mmol/L, and 2 h ≥ 8.5 mmol/L). We were not able to obtain data for women with a 2-h value between BG 7.8–8.4 mmol/L (i.e., NICE-ONLY; NICE 2015 positive and IADPSG negative). All women were treated for GDM using targets of fasting BG < 5.3 mmol/L and 1-h post prandial BG < 7.8 mmol/L respectively. Results: Of 159 women, 65 (40.9%) were NICE + IADPSG and 94 (59.1%) IADPSG-ONLY. Hypoglycaemic medication use was similar in both groups: 52.3% NICE + IADPSG, 46.8% IADPSG-ONLY, OR 1.0 (0.5–1.9). The IADPSG-ONLY group delivered later than the NICE + IADPSG group; 39.0 weeks (sd 1.4) compared to 38.2 weeks (sd 2.5), *p* value 0.02. Fewer caesarean sections occurred in IADPSG-ONLY group 30.9% vs. 52.3%, OR 0.4 (0.2–0.9). Birthweight, large for gestational age, and other neonatal complications were not significantly different between groups. Conclusions: Gestational diabetes-associated perinatal complications were similar in both groups. The IADPSG criteria detect women with evidence of ongoing hyperglycaemia who may benefit from treatment during pregnancy.

## 1. Introduction

Gestational diabetes mellitus (GDM) is defined as “glucose intolerance with onset or first recognition during pregnancy” [1,2]. It is a common complication of pregnancy with a prevalence between 2% and 40%, depending on population, method of testing, and geographic location around the world. The prevalence is increasing, secondary to rising rates of obesity, ethnic diversity, older maternal age, and changing diagnostic criteria [3]. GDM is associated with adverse pregnancy outcomes including pre-eclampsia, increased fetal growth associated with large for gestational age and macrosomia, shoulder dystocia, birth trauma, and neonatal hypoglycaemia [4,5]. Moreover, women with GDM have approximately a 50% increased risk of developing type-2 diabetes in the decade following pregnancy [6], making GDM a condition of great public health interest in the fight against the global non-communicable diseases epidemic. Despite these risks, GDM screening and diagnosis has for decades been the subject of controversy [7].

In 2015 the National Institute for Health and Clinical Excellence (NICE) updated their GDM diagnostic criteria [8,9]. This was the first statement since the 2010 International Society of Diabetes in Pregnancy Study Group (IADPSG) criteria were published. The IADPSG criteria were based on findings of the large multinational Hyperglycemia and Adverse Pregnancy Outcome (HAPO) study, designed to settle, once and for all, the ongoing controversy around the selection of diagnostic thresholds for GDM. However, as the HAPO data demonstrated a linear rather than threshold relationship between hyperglycaemia in pregnancy and adverse outcomes, GDM was defined by a group of international experts at the thresholds associated with an increase in relative risk of 1.75 for four key outcomes: primary caesarean section, neonatal hypoglycaemia, neonatal hyperinsulinaemia, and macrosomia [10,11]. The IADPSG criteria have subsequently been endorsed by WHO, FIGO (International Federation of Gynaecology and Obstetrics), the American Diabetes Association, and several other national diabetes societies. The criteria selected follow a 75 g OGTT were fasting BG ≥ to 5.1 mmol/L, 1 h BG ≥ 10.0 mmol/L and 2 h BG ≥8.5 mmol/L. However, in most populations more women will be identified with GDM when the IADPSG criteria are used, without evidence that there is any apparent benefit to them or their baby of managing them as GDM [12,13]. Therefore, the 2015 NICE guidelines recommended against IADPSG criteria, due to the lack of prospective clinical and economic evidence, instead choosing to define GDM following a 75 g OGTT as fasting BG ≥ to 5.6 mmol/L and/or a 2 h BG ≥ to 7.8 mmol/L. NICE stated that “after considering the health economic evidence, the group recommended a fasting BG of 5.6 mmol/L and a 2-hour BG of 7.8 mmol/L” although they acknowledged the limitations of the models on which they data were based [9]. 

Whilst ideally one definition of GDM would be applied to all women, controversy remains over which criteria best identifies the women at risk of pregnancy related diabetic associated maternal and perinatal complications. The objective of this study was to compare outcomes in pregnancies complicated by GDM defined by IADPSG criteria only to those defined by both the NICE 2015 and IADPSG criteria.

## 2. Materials and Methods

This was a secondary analysis of data collected for the randomised controlled trial TREAT-GDm conducted in a large tertiary hospital in Oxford, UK (registration number NCT01916694, ethical approval: NRES Berkshire ‘B’ committee, Rec reference 13/SC/0176) [14]. The trial’s primary aim was to compare smartphone-based blood glucose (BG) management with standard paper-based monitoring for women with GDM [15]. Full methodology and outcomes of the trial have been published elsewhere [15]. There were no differences in BG control or maternal or neonatal outcomes between the groups.

Pregnant women were screened for GDM based on risk factors from the NICE 2008 guidelines (BMI above 30 kg/m^2^, previous macrosomic baby weighing 4.5 kg or above, previous GDM, family history of diabetes and minority ethnic family origin with a high prevalence of diabetes) and referred for a 2-h 75 g OGTT at 16 weeks (in women with previous GDM) or between 26–28 weeks (no previous history of GDM, or if the 16 week OGTT was normal). GDM was diagnosed using the IADPSG criteria. Women with GDM were eligible if they were aged over 18 and not requiring insulin after 1 week of BG monitoring. 

For this analysis we included women with complete OGTT data (i.e., all results available for fasting, 1-h and 2-h tests). These women were retrospectively assigned to two groups: the NICE + IADPSG group (OGTT values of either fasting BG ≥ 5.6 mmol/L and/or 2 h BG ≥ 7.8 mmo/L), and the IADPSG-ONLY group (fasting BG ≥ 5.1 mmol/L, and/or 1 h BG ≥ 10.0 mmol/L and/or 2 h BG ≥ 8.5 mmol/L). 

All women were asked to test their blood glucose six times a day on at least 3 days of the week, as per the local guideline: fasting, 1-h post-breakfast, pre-lunch, 1-h post-lunch, pre-dinner, and 1-h post-dinner. The target blood glucose range was fasting BG readings ≥3.5 mmol/L and <5.3 mmol/L and 1-h postprandial BG readings less than 7.8 mmol/L. Dietetic support was offered to all women. A decision to start pharmacological treatment was made following the local trust treatment guidelines and was based on two or more readings at the same time of day above target each week despite dietary intervention [15].

We used prescription of hypoglycaemic medication (both oral agents (metformin) and insulin) as a marker of ongoing clinically significant hyperglycaemia during pregnancy. Other outcomes compared were maternal diabetes associated complications, including preeclampsia, mode of birth, gestational age at delivery, and diabetic associated neonatal complications: size of the baby (based on INTERGROWTH-21st size at birth standards), hypoglycaemia; birth trauma; admission to special care baby unit (SCBU) and a composite outcome including shoulder dystocia, birth trauma, fracture, nerve palsy or death. Detailed definitions of all outcome variables have previously been described [15]. 

Baseline binary characteristics are compared using Chi squared tests with p values, continuous variables are compared with student t tests for normally distributed data and Mann–Whitney U tests used for non-normally distributed data. Tests were considered statistically significant at *p* < 0.05. Outcome data are presented as odds ratios with 95% confidence intervals, comparing GDM associated perinatal complications between the two groups. These were calculated using binary logistic regression, adjusting for any significant differences identified from the baseline characteristics. All analysis were performed using SPSS version 21.0 (IBM Corporation, Armonk, NY, USA). 

## 3. Results

From September 2013 to June 2015, 203 women with GDM by the IADPSG criteria participated in the trial TREAT-GDm [15]. Of these women, 159 women had complete OGTT data and were therefore eligible for inclusion in this secondary analysis. Based on the OGTT result, 65 women (40.9%) would also have been classified as GDM if the NICE criteria had been used (NICE + IADPSG group). The remaining 94 women (59.1%) were detected as having GDM by the IADPSG criteria, but would not have been called GDM if the NICE-criteria had been originally used (IADPSG-ONLY group). 

Baseline characteristics of both groups are shown in Table 1. The groups were similar, with the exception women were slightly lighter in the IADPSG-ONLY group at the start of pregnancy: 82.7 kg (sd 18.7) compared to 85.7 kg (sd 22.5), and a higher proportion of women in the NICE + IADPSG group had a previous caesarean section: 54.3% compared to 29.5%.

The results of the OGTT are shown in Table 2 and the Supplementary Table S1. The 1-h test detected the most women in the study with GDM (108/159). For women in the IADPSG only group, 56/94 were diagnosed only using the 1-h result and for 56 women were diagnosed based on the 1-h BG value alone. Of the 44 women with a fasting BG value between 5.1 mmol/L to 5.5 mmol/L (i.e., below the NICE GDM fasting threshold of 5.6 mmol/L), 28 women in the IADPSG only group were diagnosed on the fasting value alone (they were negative for 1 and 2 h values). There was a correlation between BMI and fasting blood glucose. For every 1 kg/m^2^ increase in BMI, there was an increase in fasting blood glucose of 0.05 mmol/L (95% CI 0.03–0.06, *p* < 0.001). No correlation was evident between BMI and the 1 h and 2 h values.

Diabetes related maternal and neonatal outcomes are shown in Table 3. There was no difference in the proportion of women requiring hypoglycaemic medications (oral agents or insulin): 52.3% in the NICE + IADPSG and 46.8% in the IADPSG-ONLY group, OR 1.0 (95% confidence interval 0.5 to 1.9, Table 3). Women requiring hypoglycaemic medication, regardless of allocation group, experienced more pregnancy complications with an increased risk of caesarean section, OR 1.9 (1.0–3.6), LGA, 2.2 (1.0–4.8), earlier gestational age at delivery (38 vs. 39 weeks, *p* = 0.019), and a borderline increased risk of birthweight > 4 kg, OR 2.5 (0.9–7.0). When considering outcomes for those using hypoglycaemic medications by IADPSG/NICE subgroups, numbers for most complications are very small, and for most outcomes no significant differences were found, however, there was an increase in LGA risk amongst women who took medication in the IADPSG only group compared to those not on medication, OR 3.5 (1.2–10.6). 

The proportion of women who developed preeclampsia did not differ between the groups. There was, however, a difference in the gestational age of delivery, with the NICE + IADPSG women delivering 5 days earlier than the IADPSG-ONLY: 38.2 weeks (sd 2.5) compared to 39.0 weeks (sd 1.4), *p* = 0.02. After accounting for the baseline difference in the proportion of women with a previous caesarean section, IADPSG-ONLY women were less likely to be delivered in this pregnancy by caesarean, OR 0.4 (0.2–0.9, *p* = 0.03). 

There were no significant differences in other maternal outcomes such as postpartum haemorrhage or perineal trauma. 

There were no stillbirths or neonatal deaths within the cohort. Rates of neonatal complications associated with diabetes did not significantly differ between the two groups, with a similar proportion of babies in each group suffering a severe outcome (shoulder dystocia and/or birth trauma); 3.2% in the IADPSG-ONLY group and 1.5% in the NICE+IADPSG group. Birthweights were similar between the groups and there were no significant differences in the proportion of babies born LGA (> 90th centile) or macrosomic (birthweight > 4 kg). 

## 4. Discussion

Our results add to the growing body of literature that support the use of the IADPSG criteria, and specifically the ability of the 1-h threshold of the OGTT to identify a group of women with clinical evidence of persistent hyperglycaemia who experience GDM-associated adverse outcomes [12,16]. To our knowledge, this is one of the first publications to investigate the comparison between women diagnosed and treated for GDM based on either the NICE 2015 criteria or IADPSG criteria, contributing to the on-going debate about the most appropriate GDM diagnostic criteria to use in the UK. 

Whilst it is striking in this study that the IADPSG-ONLY group comprised a greater number of women (59% of the total), we were not able to determine how many additional women (if any) in our population were diagnosed using the IADPSG criteria compared to if we had of applied the full NICE-2015 criteria. That is; the group of women who are not included in the IADPSG criteria the group with a 2-h value of the OGTT between 7.8–8.4 mmol/L. With regards to the evidence on which NICE made its recommendations for the 2 h value threshold, NICE states it included the highest quality data from a single population of women. They believe that the fasting value of 5.6 mmol/L and 2 h value of 7.8 mmol/L were reasonable as these criteria have cost effective evidence to support their use [9].

Persistent hyperglycaemia, which we defined in this study as meeting the clinical indication in our institution for hypoglycaemia medication, was equally apparent in both groups. In the NICE+IADPSG group 52.3% of women required treatment compared to 46.8% in the IADPSG-ONLY group. The proportion of LGA babies was also similar in both groups, affecting approximately 1 in 5 babies, consistent with reports in gestational diabetic populations [17]. In our study, high rates of neonatal hypoglycaemia were observed in both groups (1 in 3 babies). Neonatal hypoglycaemia can be a serious complication if unrecognised. National guidelines recommend routine neonatal blood glucose monitoring for 24 h in babies born to women with diabetes. 

The strengths of this study are the completeness and accuracy of outcome data and that the outcomes are reflective of actual practice in a busy public hospital in the UK. However, we acknowledge the limitations. Firstly, the study is small and therefore prone to type 2 error (i.e., statistically relevant differences may be overlooked) and we lack adequate power to show differences in less frequent complications. We were not able to perform longer term follow up on these women, and it may be that there are differences in the risk of type 2 diabetes between the two criteria. Future type-2 diabetes risk of these women needs to also be considered before a full cost-benefit analysis of GDM screening and diagnosis can be made, as much of the cost-benefit may come from future prevention of type 2 diabetes [18]. 

There is currently no randomised data comparing the IADPSG criteria compared to the NICE criteria. However, we demonstrate in our observational study that the IADPSG criteria detect a group of women with evidence of persistent hyperglycaemia who experience similar rates of associated pregnancy complications to those also diagnosed by the NICE criteria. This confirms observations other groups have made that women who screen positive for GDM according to the IADPSG criteria have increased rates of complications compared to the background population [19]. What we have demonstrated in addition to this is that, when treated for gestational diabetes, the complication rates are similar to women classified by NICE. Whilst we cannot infer from our data that treatment improved outcomes for these women, other trials have demonstrated that there is benefit in treating women with demonstrated ongoing hyperglycaemia in pregnancy to prevent adverse perinatal outcomes [4]. The controversy remains around which diagnostic test should be used to determine which women will be monitored for hyperglycaemia and whether universal screening of GDM for all pregnant women should be adopted, as universal screening has been demonstrated to improve perinatal outcomes in women who were treated for GDM [5].

Pregnancy provides the ideal opportunity to engage women in healthy initiatives to improve future health [20]. It could therefore be argued that the IADPSG criteria should be adopted universally, so as not to delay or omit treating these women at increased risk of pregnancy and longer-term problems. We believe that more studies to identify OGTT thresholds for diagnosis of GDM are not needed; rather research should focus now on strategies for prevention, and for those women who do develop hyperglycemia in pregnancy, the focus must be on developing sustainable and clinically effective management strategies that also improve lifelong health in the mother and child. 

## Figures and Tables

**Table 1:** Baseline characteristics of participants according to group, *N* = 159.

Characteristic	IADPSG Only *N* = (%)	NICE + IADPSG *N* = (%)	*p* Value
	*N* = 94 (59.1)	*N* = 65 (40.9)	
Maternal age (years)			
Mean (sd)	33.6 (5.5)	33.5 (5.7)	1.0
Parity	*N* = 94	*N* = 65	
0	33 (35.1)	30 (46.2)	0.7
1	34 (36.2)	23 (35.4)	
2 or more	27 (28.7)	12 (18.5)	
Height in meters	*N* = 94	*N* = 65	0.5
Mean (sd)	1.63 (0.07)	1.63 (0.08)	
Weight in kg	*N* = 94	*N* = 65	
Mean (sd)	82.7 (18.7)	85.7 (22.5)	0.04
BMI (m/kg^2^)	*N* = 94	*N* = 65	
Median (IQR)	30.8 (22.8–38.8)	31.9 (20.0–43.8)	0.27
Educational level	*N* = 92	*N* = 65	
GCSE or less	21 (22.8)	19 (29.2)	0.6
A Level	26 (28.3)	15 (23.1)	
University	45 (48.9)	31 (47.7)	
Ethnicity	*N* = 93	*N* = 65	
White	72 (77.4)	51 (78.5)	1.0
South Asian	10 (10.8)	8 (12.3)	
Other	11 (11.8)	6 (9.2)	
Smoker	*N* = 94	*N* = 65	
Yes	3 (3.2)	4 (6.2)	0.4
Chronic Hypertension	*N* = 93	*N* = 65	
Yes	5 (5.4)	1 (1.5)	0.2
1st-degree relative with diabetes	*N* = 92	*N* = 63	
Yes	35 (38.0)	28 (44.4)	0.3
Allocation group in RCT *	*N* = 94	*N* = 65	
Control	50 (53.2)	31 (47.7)	0.5
Intervention	44 (46.8)	34 (52.3)	
Previous baby > 4.5 kg	*N* = 61	*N* = 35	
Yes	7 (11.5)	1 (2.9)	0.2
Previous GDM	*N* = 61	*N* = 35	
Yes	6 (9.8)	9 (25.7)	0.08
Previous caesarean section	*N* = 61	*N* = 35	
Yes	18 (29.5)	19 (54.3)	0.03

* TREAT-GDm^15^.

**Table 2:** Distribution of blood glucose values from the OGTT. All women in the study were positive for GDM by one or more values of the IADPSG criteria. A subgroup of these women were also positive for GDM applying the NICE 2015 criteria. Women may have 1, 2, or 3 positive values (see Supplementary table for detailed breakdown).

OGTT Value		All Women *N* = 159	IADPSG Only *N* = 94	NICE + IADPSG *N* = 65
Fasting				
	<5.1	81	56	25
	5.1–5.5	44	38	6
	≥5.6 *	34	-	34
1-h				
	<10.0	51	28	23
	≥10.0	108	66	42
2-h				
	<7.8	116	94	22
	7.8–8.4	11	0	11
	≥8.5	32	-	32

* NICE threshold for fasting blood glucose level.

**Table 3:** A comparison of maternal and neonatal outcomes in women with GDM identified by NICE or IADPSG criteria.

	IADPSG Only *N* (%)	NICE + IADPSG *N* (%)	OR 95% CI *	*p* Value
*Maternal outcomes*				
Hypoglycaemic medication use	*N* = 94	*N* = 65		
Yes	44 (46.8)	34(52.3)	1.0 (0.5–1.9)	0.9
Preeclampsia	*N* = 94	*N* = 64		
	10 (10.6)	2 (3.1)	4.2 (0.9–21.0)	0.08
Gestational age at delivery in weeks	*N* = 94	*N* = 65		
mean (sd)	39.0 (1.4)	38.2 (2.5)		0.02
Spontaneous onset of labour	*N* = 94	*N* = 65		
Yes	22 (23.4)	16 (24.6)	0.8 (0.4–1.7)	0.5
Caesarean section	*N* = 94	*N* = 65		
Yes	29 (30.9)	34 (52.3)	0.4 (0.2–0.9)	0.03
PPH > 500 mls	*N* = 93	*N* = 64		
Yes	41 (44.1)	32 (50.0)	0.9 (0.5–1.7)	0.7
Major perineal trauma	*N* = 92	*N* = 65		
Yes	3 (3.3%)	0 (0%)	-	
*Neonatal Outcomes*				
Birth weight in grams	*N* = 94	*N* = 64		
Mean (sd)	3333 (652)	3438 (491)		0.2
Large for gestational age (birthweight > 90th centile)	*N* = 93	*N* = 61		
Yes	23 (24.7)	13 (21.3)	1.3 (0.6–2.9)	0.5
Macrosomia (>4 kg)	*N* = 94	*N* = 64		
Yes	12 (12.8)	7 (10.9)	1.3 (0.5–3.6)	0.6
Neonatal hypoglycaemia	*N* = 90	*N* = 58		
Yes	25 (27.8)	19 (32.8)	0.8 (0.4–1.7)	0.6
Admission to SCBU	*N* = 93	*N* = 63		
Yes	9 (9.7)	4 (6.3)	1.7 (0.5–5.9)	0.4
Composite severe neonatal outcome **	*N* = 94	*N* = 64		
Yes	3 (3.2)	1 (1.5)	2.2 (0.2–22.6)	0.5

* all OR adjusted for booking weight and previous caesarean section; ** Shoulder dystocia and/or birth trauma, nerve palsy, fracture, death; Neonatal hypoglycaemia is defined as serum blood glucose level of <1.5 mmol/L or requiring SCBU admission for feeding.

## Supplementary Materials

The following are available online at http://www.mdpi.com/2077-0383/7/10/376/s1, Table S1: Breakdown of OGTT results in mmol/L. Colour code: Blue = negative on both criteria; Yellow = IADPSG only; green = positive on NICE criteria. * As all women were positive on the IADPSG criteria in this study there were no women positive on the 2-hour value alone for NICE.

## Novelty Statement

This study adds to the debate regarding diagnostic criteria for GDM, making a case to continue using the IADPSG criteria as opposed to the NICE 2015 criteria so as not to overlook women with GDM associated maternal and neonatal complications.
