# Supplementary material for: Clinical Implications of the NICE 2015 Criteria for Gestational Diabetes Mellitus
Source: J Clin Med. 2018 Oct 22;7(10):376. doi: 10.3390/jcm7100376 (PMC6209967; doi:10.3390/jcm7100376)
Supplement: Supplementary file 1 [file jcm-07-00376-s001.pdf]

Supplementary Materials

Table S1. Breakdown of OGTT results in mmol/L. Colour code: Blue = negative on both criteria; Yellow = IADPSG only; green = positive on NICE criteria. *As all women were positive on the IADPSG criteria in this study there were no women positive on the 2-hour value alone for NICE.

Fasting			1-hour		2-hour			Total	IAPDSG -ONLY	NICE + IADPSG
≤5.0	5.1-5.5	≥5.6	<10.0	≥10.0	<7.7	7.8-8.4	≥8.5			
✓			✓				✓	9		9
✓			✓			✓		0*		
✓				✓	✓			56	56	
✓				✓		✓		4		4
✓				✓			✓	12		12
	✓		✓		✓			28	28	
	✓		✓			✓		1		1
	✓		✓				✓	2		2
	✓			✓	✓			10	10	
	✓			✓		✓		1		1
	✓			✓			✓	2		2
		✓	✓		✓			8		8
		✓	✓			✓		1		1
		✓	✓				✓	2		2
		✓		✓	✓			14		14
		✓		✓		✓		4		4
		✓		✓			✓	5		5
**Total**								**159**	**94**	**65**
